# Engaging patients and citizens in digital health technology development through the virtual space

**DOI:** 10.3389/fmedt.2022.958571

**Published:** 2022-11-25

**Authors:** Romina Helena Barony Sanchez, Laurie-Ann Bergeron-Drolet, Maxime Sasseville, Marie-Pierre Gagnon

**Affiliations:** ^1^ VITAM Research Center on Sustainable Health, CIUSSS Capitale-Nationale, Laval University, Quebec City, QC, Canada; ^2^ Faculty of Medicine, Laval University, Quebec City, QC, Canada; ^3^ The International Observatory on the Societal Impacts of AI and Digital Technology, Quebec City, QC, Canada; ^4^ Faculty of Nursing, Laval University, Quebec City, QC, Canada

**Keywords:** digital health, patient and citizen engagement, underrepresented groups, virtual collaboration, co-development.

## Abstract

Digital technologies are increasingly empowering individuals to take charge of their health and improve their well-being. However, there are disparities in access related to demographic, economic, and sociocultural factors that result in exclusion from the use of digital technologies for different groups of the population. The development of digital technology in health is a powerful lever for improving care and services, but also brings risks for certain users in vulnerable situations. Increased digital health inequalities are associated with limited digital literacy, lack of interest, and low levels of self-efficacy in using technology. In the context of the COVID-19 pandemic and post-pandemic healthcare systems, the leap to digital is essential. To foster responsible innovation and optimal use of digital health by all, including vulnerable groups, we propose that patient and citizen engagement must be an essential component of the research strategy. Patient partners will define expectations and establish research priorities using their experiential knowledge, while benefiting from rich exposure to the research process to increase their self-efficacy and digital literacy. We will support this proposition with an operationalised example aiming to implement a Virtual Community of Patients and Citizens Partners (COMVIP), a digital tool co-created with patients and public experts, as active team members in research. Founded on the principles of equity, diversity and inclusion, this base of citizen expertise will assemble individuals from different backgrounds and literacy levels living in vulnerable situations to acquire knowledge, and share their experiences, while contributing actively in the co-development of innovative strategies and health technology assessment.

## Introduction

Digital tools have developed rapidly over the past two decades and are being used increasingly in healthcare as they are associated with improved well-being and health (1, 2). They facilitate clinician-patient collaboration and encourage patients to interact and participate actively in their care. Furthermore, the active involvement of patients in their care process allows for improved clinical outcomes and health services quality (3, 4). The use of digital tools in healthcare is essentially changing the way clinicians deliver care and inform patients about their health (3, 4). Digital tools have the ability to easily adapt to change and to people's profiles because of their versatility and dynamism (3). However, social inequalities cause significant disparities in access to Canadian health services and digital technologies (5–10). Vulnerable populations are often underserved by telecommunications services and are deprived of optimal access to employment, education and health and social services (5–9). They are more prone to chronic diseases, social isolation, lower socio-economic status, lower education and harmful health behaviours such as smoking (8–11). Digital technologies help break isolation of older people living alone, however their use is often hindered by a lack of access and familiarity with their use (7, 8). Cultural minorities, including Indigenous populations, are underrepresented in digital heath data, which causes biases against them when using these tools (12). They also have difficulty searching for, and understanding health information and services using digital tools (8, 9).

**Figure 1:**
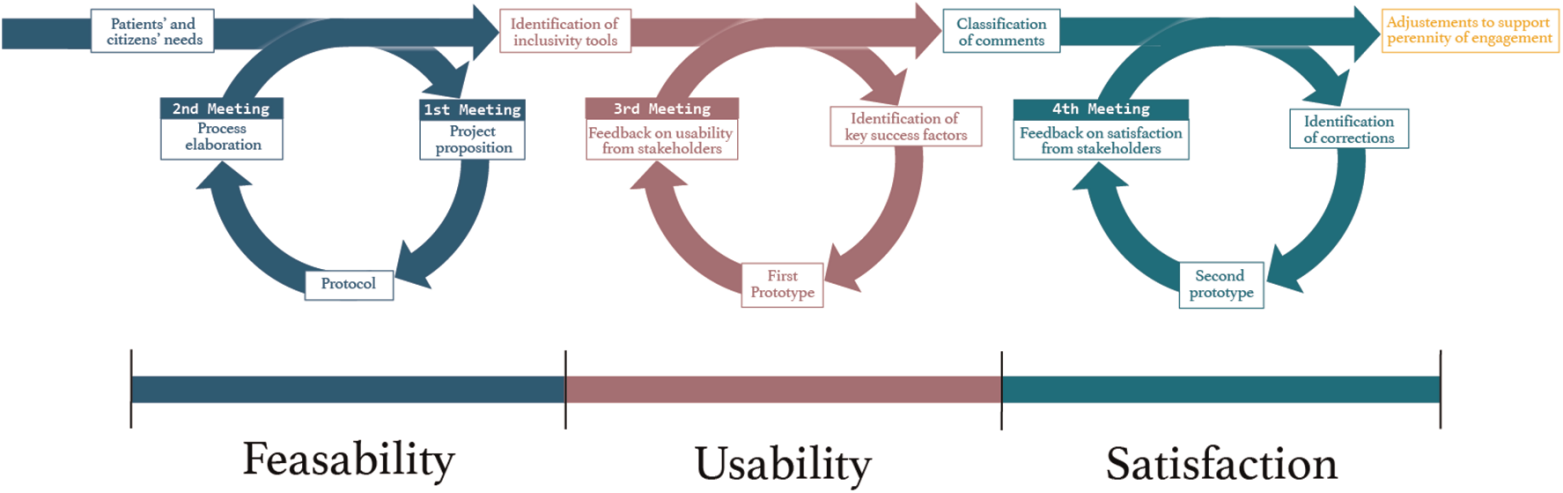
The COMVIP project Co-creation process.

The COVID-19 pandemic accelerated use and adoption of digital health technologies, while showing the ability of digital solutions to meet many of the population needs (4). However, the access and use of digital health innovations by specific population groups in vulnerable situations remain limited. These digital health inequities may be associated with a range of factors. Among others, certain demographic, economic, and sociocultural characteristics, limited computer literacy, lack of interest, and low levels of technology self-efficacy are barriers to digital health tools (4, 13). In addition, digital health technology solutions are proving inadequate to meet the specific needs of vulnerable groups as they are often designed without their insight and experiences (14, 15). People typically underrepresented in digital health solutions include, older adults, people living with disabilities, youth in difficulty, people from cultural minorities, and Indigenous people (Figure 1).

Underrepresented groups do not fully benefit from the opportunities offered by digital technologies to access health services (13). This article presents the concept of patient and public engagement in the development of a digital health technology platform and an example of its application using an innovative co-creation methodology.

## Patient engagement towards digital technologies

Meaningful patients and citizens' engagement in the research process is of utmost importance for successful implementation and application of research results (16). To achieve the goal of active patient and public engagement, it is imperative to foster an inclusive climate in which all those involved in the research process understand the value of shared experiential knowledge and (16). Patients and public partners can take meaningful and key roles in research by supporting access to peer networks and difficult to reach groups and peer-to-peer recruitment (16). This can also apply to the development of useful and adaptable digital technology intended for patients. To increase the adoption of digital tools, it becomes essential to enhance and encourage users' engagement as active participants in technologies co-creation. Meaningful engagement leads to an improved understanding of their experiences, preferences, needs, as well as potential limitations of digital tools (13, 14, 17).

For a digital technology to be useful and effective, it requires for its features to have the potential to attract, adapt and actively immerse the user in its content (18, 19). However, several studies show that the effectiveness of technology interventions may be limited by inappropriate use by the intended users, leading to the abandonment or rejection of the tool over time (19, 20). Technology design, appearance, and functionality are thus important precursors to user engagement (21). These factors encourage an affective and behavioral connection to the proposed tool (22). Despite the lack of academic consensus, user engagement in health appears to be a multidimensional construct including cognitive, behavioral and affective components that allow the user to effectively adopt a digital tool (21, 22).

Despite the growing presence of tools to evaluate engagement, the use of inclusive approaches from a variety of fields is still needed to allow their application to diverse groups (23). These tools should combine both quantitative and qualitative assessment of users' perspectives in the design and use of technologies (23–25). As such, the design process of The Virtual Community of Patients and Citizens Partners (COMVIP) strived to integrate patients and users' perspectives, from the conception of the research process to the development and testing of the platform to ensure maximum retention, adaptability and satisfaction.

The digital tool created will allow for its continuous design evolution and options' adaptation according to user experience and patients and public preferences, therefore, maintaining engagement over time. By monitoring patients and public usage activity within the platform and openly discussing barriers and needs, COMVIP may adapt more effectively to users. As such, it allows for the continuity and sustainability of research projects and digital technology development, which are quality indicators of engagement (26). Thus, COMVIP seeks to adjust to the current and future needs of its intended users by presenting an adaptable, customizable, informative and inclusive navigation environment.

## Answering a collective need: the virtual community of patients and citizens (COMVIP)

To support the efforts towards patient and public participation in the development of digital technologies in research and health technology assessment, the idea of a virtual platform for knowledge and experience sharing was born. This feasibility project aims to implement and evaluate COMVIP, an innovative intervention co-constructed by patient and citizen partners, researchers and community organizations. It is founded on deliberative approaches (27) and Canadian Institutes for Health Research (CIHR) Patient Engagement Framework's principles of equity, diversity, support, mutual respect, co-building and inclusion (16). A partnership with patients and citizens has been established for the co-creation process depicted in this article. The team can count on the active involvement of patients and citizens as they are considered members of the research team and collaborate in each step of the research project. Their active involvement will provide support through shared experiences in care and use of technology. Patients and citizens are considered experts in the different research methods and activities, and will participate in the writing of the research protocols, planning of research methods and implementation and dissemination of the research results. COMVIP will provide an opportunity to gain knowledge, share experiences, and support team members in the process of digital ownership and empowerment.

The objectives of this project were to:

(1) foster meaningful engagement of patients and citizens in the development of digital technologies in health;
(2) increase digital health literacy and level of confidence in the use of health technologies;
(3) identify barriers of effective digital health tools use.

The project will contribute to the digital transformation in health and health technology assessment by involving users in tools development. It will promote strategies aimed at under-represented groups inclusion in digital health projects in Canada and elsewhere in the world and will guide practice implementation to encourage user empowerment. Digital technologies can be seen as a lever for rapid access to care and services and better resource utilization. The inclusion of end users in the design of digital health projects favors adoption of digital tools and it is a recognized approach for a responsible innovation process (2, 28). Patient and citizen partners will be invited to define their expectations and establish their priorities in the use of digital solutions that will then be co-developed and tested in subsequent research projects.

Patients and citizen partners come from a variety of backgrounds (community organizations, Indigenous peoples, immigrants, people living with specific health conditions) and are committed to the development of COMVIP. To them, this platform is a lever for making their voices heard and influencing the digital transformation of the healthcare system. For the team members who come from the domains of academic research and the development of digital solutions, COMVIP responds to a need for access to the experiential knowledge of people who could benefit from the use of digital tools in health, but who often remain difficult to reach.

The project inspired the creation of an innovative and iterative methodology adapted to the existing circumstances associated with COVID-19 that limited physical interactions. This co-construction model allowed team members to cooperate effectively and begin the COMVIP platform design process in a virtual setting.

## An innovative and iterative methodology: A co-creation process

The research team is composed of university professors, health professionals, graduate students and experts in the fields of medicine, nursing, public health, social sciences, education, ethics, marketing, mathematics, computer science and AI from different institutions in the Province of Quebec such as Université Laval, the Université du Québec à Montréal (UQAM), the Université de Montréal, the Université du Québec à Trois-Rivières and the Centre de recherche universitaire sur les jeunes et les familles (CRUJeF). The research team also counts on the active involvement of expert patients and partners from the Unité de soutien système de santé apprenant Québec and community organizations such as the Centre d'amitié autochtone du Québec, the Association des étudiantes et étudiants de l'Université du 3e âge de Québec (AEUTAQ), the Regroupement des organismes de personnes handicapées de la région de la Capitale-National ROP03) and the Service de Référence en Périnatalité pour les Femmes Immigrantes de Québec, among others.

We opted to use a collaborative application and conferencing tool to work remotely on the same documents, allowing sharing real time advancement in the project with all involved. A first virtual meeting took place on February 2021 to officially launch the COMVIP project. The senior researcher of the project ascertained beforehand the ability of all team members to access a virtual meeting and interact efficiently. Since sanitary measures were implemented in March 2020, attendees were comfortable with virtual gatherings. They were considered increasingly useful for social engagements (work meetings, webinars, concerts, family meetings, public hearings, etc.).

As noted by Rasburn and colleagues (29), there are numerous benefits to having virtual meetings as a working tool. It enhances accessibility, by removing barriers that could have prevented participation; inclusivity, by allowing participants to control and adapt freely the conference tool's settings (lighting, speaker sound, microphone sound, camera) and feel comforted by being in their own environment; and transparency, by allowing more people to attend and observe gatherings (28). Virtual meetings enable participants to attend team gatherings from home thus, reducing travel time, costs, fatigue and recovery time (29). Moreover, they are easily accessible for people who live further from the physical location of the meeting, who have caring responsibilities, work engagements or other commitments (28). Consequently, it enables the research team to invite more people and have a broader range of perspectives (28). The research team adapted to digital literacy levels by actively listening to attendees' problems and making themselves available to solve any technical difficulties before officially starting the gatherings.

A co-creation process was implemented to ensure that patients and citizens' perspectives would be fully integrated in the platform development, since it is destined to be used by them and to benefit their associated population. An inclusive approach to co-creation and co-production enhance patients and public engagement in projects in line with their priorities and interests, and facilitates the implementation process (30, 31). The research team made a great effort to ensure and stimulate the collaboration and participation of everyone in the discussion, encourage them to share their personal knowledge, consider others' opinions and use group tensions to enhance creativity and productivity (32, 33). This allowed all members to express themselves in a respectful and inclusive environment that favors successful patient engagement (26). In a co-creation process, it is suggested that stakeholders go through an iterative process (34). Thus, the research project adhered to a cycle of co-creation consisting of four steps: exploration of solutions and avenues; decision-making about what ideas should be kept; creation of a prototype and evaluation of the prototype (34).

Patients and other stakeholders came mostly from Quebec City and Montreal and were involved in the conception of COMVIP from the beginning. During the first meeting, which took place on February 8, 2021, the research team discussed about the objectives of the research project and the platform's intent, so that all involved agreed upon the aims and develop a shared purpose which is a key patient engagement quality criterion (26). Then, team members could comment, discuss limits and barriers from their perspective and propose opportunities and ideas to the research team. All team subgroups were present and represented by at least one member during the gathering (patient and citizen partners, community organization representatives, researchers working on patient-centered care projects and team members leading the project) to reach stakeholders' representativeness (26). The first meeting allowed to explore the feasibility of the COMVIP platform with intended users and stakeholders. During the second team meeting, which took place on April 20, 2021, participants identified together content and functionalities to be added to the prototype according to users' identified needs. From April 2021 to May 2022, the research team developed a platform prototype with the help of a graphic designer and web developer. The prototype creation was inspired by Ruel and Allaire's guide on accessible information (35) and the Web Content Accessibility Guidelines (WCAG) international standard for people with disabilities from the W3C Web Accessibility Initiative (36). The result of the co-creation process was the development of a single-paged and easy-to-use platform prototype. It offers simplified menus for rapid and easy access to specific content (e.g.,: forums, courses, profile), font size modification icons for better reading experience, vivid colors adapted to the visually impaired and color-blind, inclusive images representing the variety of peoples and cultures that make up the population, easy access to projects’ description and a space presenting the name, photo and contact information of the different members of the COMVIP team.

The COMVIP prototype was presented to stakeholders during a third team meeting which took place on May 12^th^, 2022. The leading researchers invited everyone to comment, give their opinion, and propose possible improvements. The prototype was positively welcomed by patients and citizens partners and researchers. New comments and propositions for better user experience and easier navigation were gathered from their feedback, such as the development of user stories, the inclusion of an introductory user descriptive video clip, standardized project profile resumes and the addition of useful project links. Thus far, three meetings have taken place either online or in hybrid mode and lasted two hours each. An average of twenty people attended each meeting and every subgroup was represented by at least one person or more. All team members from professorial and experts in the different fields previously mentioned were present during the meetings.

**Figure 2:**
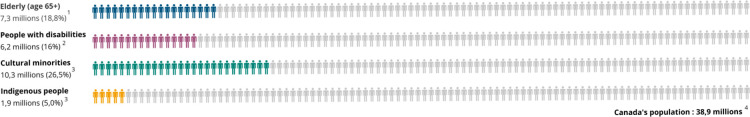
A portrait of Canadian underrepresented groups living in vulnerable situations. 1. Statistics Canada. (2022). *Demographic estimates by age and sex, provinces and territories: Interactive dashboard* [Data visualization tool]. Ottawa. Released July 1, 2022. https://www.statcan.gc.ca/en/subjects-start/older_adults_and_population_aging 2. Statistics Canada. (2019). *Persons with disabilities and COVID-19* [Infographic]. Ottawa. Released July 6, 2020. https://www150.statcan.gc.ca/n1/pub/11-627-m/11-627-m2020040-eng.htm 3. Statistics Canada. (2022). *Census Profile*. *2021 Census of Population*. Statistics Canada Catalogue no.98-316-X2021001. Ottawa. Released October 26, 2022. https://www12.statcan.gc.ca/census-recensement/2021/dp-pd/prof/index.cfm?Lang=E 4. Statistics Canada. (2022). *Canada's population estimates: Age and Sex, July 1 2022* [pdf]. Ottawa. Released September 28, 2022. https://www150.statcan.gc.ca/n1/daily-quotidien/220928/dq220928c-eng.pdf

All the different opinions and propositions will be reviewed according to two criteria: significance and feasibility. This process will guide decision choices about which propositions to be added to the platform. The feedback obtained from the meeting helped understand the usability of the platform and the need for minor corrections and additions. Finally, the research team will conduct an improvement iteration of the platform according to feedback. A fourth meeting will be organized in order to present the final version of the COMVIP platform and present the changes made to team members in order to evaluate their satisfaction. By the end of the project, the group should have come through three steps of the cycle of co-creation: feasibility, usability and satisfaction of stakeholders (Figure 2).

## Discussion

Through online workshops and showcases, people from various backgrounds can engage in the co-development of digital health solutions adapted to their needs. According to CIHR Patient Engagement Framework (16), successful patient engagement incorporates inclusive mechanisms and processes that allow patient and public involvement at all levels of the research process, a multi-way capacity building that promotes the development of stakeholders' capacities and a safe environment for open interactions and effective teamwork, a multi-way collaboration and communication by fostering mutual respect, an experiential knowledge of stakeholders that is valued as evidence and translated, collaborative methods of research that are inclusive and recognizes a diversity of patients, and a shared sense of purpose that allows stakeholders to work together towards a common and stay informed about research outcomes. Guided by this framework, we can concede that COMVIP supports patient and public engagement successfully in its research methods and co-creation design process. This project effectively empowers and includes stakeholders through every research and development stage while valuing stakeholders' key role through shared personal experiences and appreciation.

The participants engagement towards COMVIP will be key to its successful adoption, implementation and sustained use by stakeholders and public.

Once officially launched, COMVIP will help develop knowledge about the needs and challenges of vulnerable groups with respect to their acceptability of digital technology, beyond technical considerations, and about the factors that can promote digital health literacy among these groups. In addition, this project will help develop knowledge on the conditions that promote the commitment and continued involvement of patients and citizen partners in this type of research, as well as on the impact of their involvement in the development of digital solutions in health and social services.

## Data Availability

The raw data supporting the conclusions of this article will be made available by the authors, without undue reservation.
